# Healing of Fatigue Crack in 1045 Steel by Using Eddy Current Treatment

**DOI:** 10.3390/ma9080641

**Published:** 2016-07-29

**Authors:** Chuan Yang, Wenchen Xu, Bin Guo, Debin Shan, Jian Zhang

**Affiliations:** 1 School of Materials Science and Engineering & National Key Laboratory for Precision Hot Processing of Metals, Harbin Institute of Technology, Harbin 150001, China; yangchhit@gmail.com (C.Y.); bguo@hit.edu.cn (B.G.); shandebin@hit.edu.cn (D.S.); 2 State Key Laboratory of Advanced Welding and Joining, Harbin Institute of Technology, Harbin 150001, China; zhangjianhit@126.com

**Keywords:** fatigue crack, healing, eddy current

## Abstract

In order to investigate the methods to heal fatigue cracks in metals, tubular specimens of 1045 steel with axial and radial fatigue cracks were treated under the eddy current. The optical microscope was employed to examine the change of fatigue cracks of specimens before and after the eddy current treatment. The results show that the fatigue cracks along the axial direction of the specimen could be healed effectively in the fatigue crack initiation zone and the crack tip zone under the eddy current treatment, and the healing could occur within a very short time. The voltage breakdown and the transient thermal compressive stress caused by the detouring of eddy current around the fatigue crack were the main factors contributing to the healing in the fatigue crack initiation zone and the crack tip zone, respectively. Eddy current treatment may be a novel and effective method for crack healing.

## 1. Introduction

Fatigue failure is one of the most important failure modes for structural components working under cyclic loading. Repetition of these cycles may cause the microcracks to initiate, propagate, and merge into oversized cracks and thus lead to a fracture, decreasing the service life of structural components. In order to increase the fatigue resistance and elongate the service life, it is necessary to develop effective methods to heal the microcrack or prevent the microcrack propagation since a very small crack may be healed if the maximum temperature of heat treatment is sufficient to bring in the atoms on either side of the crack to fill the crack [1]. Until now, many techniques have been employed to heal cracks, such as SMA reinforced composites [2], healing agent capsule reinforced composites [3], electroplating [4], heat treatment [5], hot plastic deformation [6], and electropulsing treatment; electropulsing treatment is a promising method for crack healing in metallic materials since it can detect the cracks automatically, and the effect of the repair on uninjured parts is small [7,8].

An earlier study was conducted by V.M. Finkel et al. [9], which indicated that cracks in silicon iron specimens had a tendency to self-repair, and the failure of specimens could be halted after the specimens were treated using the current pulses. V.V. Levitin and S.V. Loskutov [10] investigated the influence of strong current pulses on titanium alloy, and the investigation indicated the fatigue resistance of titanium alloy increased due to the healing of crystal lattice defects. Zhou et al. [11,12] demonstrated that when different cracks in 1045 steel were treated by applying a high density electric pulse, the crack tip could be healed while the original microstructure in the vicinity of crack was maintained during the healing treatment. These different cracks included pre-cracks, fatigue cracks, and quenched cracks. Yang Ju et al. [13,14] found that fatigue cracks in stainless steel could also be healed after high density electropulsing treatment, and the crack closure and the bridging between the surfaces of a crack occurred around the vicinity of the crack tip, which may be related to the local disappearance of slip bands and the decrease of slip height on the surface of specimens. Tao Yu, De Wei, Gang Wang and Hongchao Zhang [15] also studied the influence of electropulsing treatment on the crack healing in SUS304 stainless steel, and they found that the width of crack decreased gradually from the center to both sides of the crack after seven times electropulsing discharge treatments. The mechanical properties of aluminum alloy and copper alloy subjected to short duration current pulses were studied by F. Gallo et al; their results indicate that the yield stress dropped while the elongation and plastic strain increased after the current pulse experiment [16,17].

According to previous studies, the crack healing is related to the heat effect generated by electric current flowing around the crack [10,11,12,13]. The eddy current consists of loops of electric current induced within conductors by changing the magnetic field in the conductor, due to Faraday’s law of electromagnetic induction [18]. In other words, the eddy current is a kind of electric current different from the pulse current, so the eddy current probably has the potential to heal the cracks. Moreover, the eddy current is relatively safer and more convenient to handle tubular specimens than the high density pulse current. In this study, the fatigue cracks of tubular specimens of 1045 steel were treated by employing an eddy current induced by changing the magnetic field; the eddy current could be applied in simply and in safe conditions. The evolution of fatigue cracks was analyzed to examine the effect of eddy current on fatigue crack healing.

## 2. Materials and Methods

**Figure 1:**
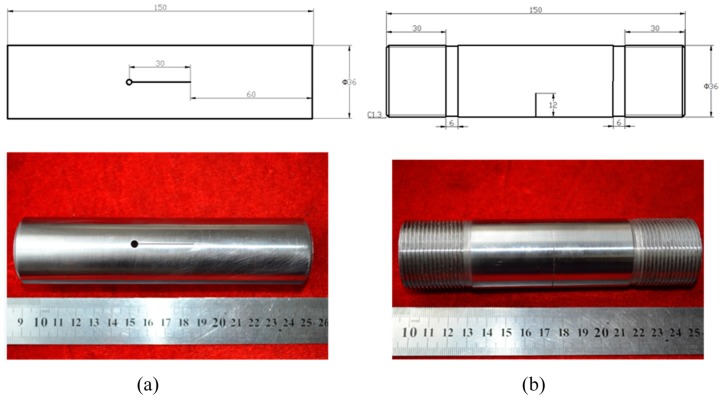
Schematics and photographs of specimens: (**a**) Group A; (**b**) Group B. (unit: mm).

The tubular specimens were cut from a 1045 steel tube and machined to 36 mm in outer diameter, 3 mm in thickness and 150 mm in height. Two types of cutting line were created in the tubular specimens through wire electrical discharge machining (WEDM, Ren Guang CNC Equipment Co Ltd, Suzhou, China), one along the axial direction and the other along the radial direction of the specimen, as shown in Figure 1. A cutting line 30 mm in length was created along the axial direction of the tubular specimen in group A, while a cutting line 12 mm in length was created along the radial direction of the tubular specimen in group B. Each group consisted of three specimens for different durations of the eddy current treatment. In order to introduce the fatigue crack, the fatigue tests were conducted at room temperature in atmosphere under the condition of dynamic load with a MTS 890 Axial/Torsional Test System (MTS Systems Corporation, Eden Prairie, MN, USA). All of the tests in group A were carried out at a stress ratio of *R* = 0.13, a maximum force of *F*_max_ = 53 KN and a frequency of ƒ = 10 Hz for 10^4^ cycle, while all of the tests in group B were performed at a stress ratio of *R* = 0.14, a maximum force of *F*_max_ = 21 KN and a frequency of ƒ = 10 Hz for 5 × 10^3^ cycle. The fatigue cracks initiated and propagated along the cutting lines during the fatigue tests. The morphologies of the fatigue cracks before and after the eddy current treatment were observed on an OLYPUS optical microscope and the fatigue crack photographs taken through the OLYMPUS optical microscope were stitched together to get full views of the fatigue cracks.

**Figure 2:**
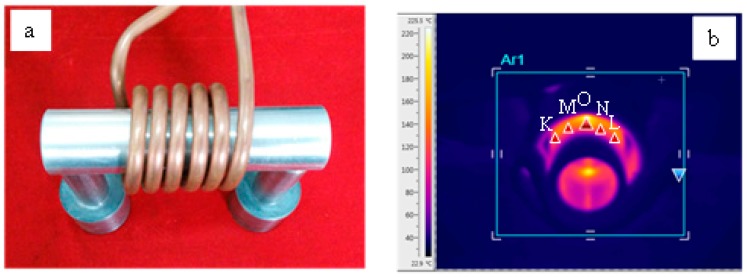

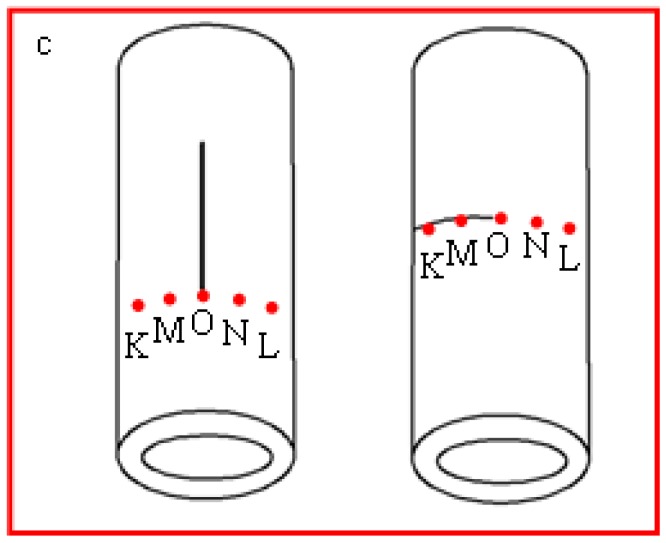
Photographs of the copper coil and specimen: (**a**) specimen within copper coils; (**b**) infrared temperature image of the surface of specimen; (**c**) measurement points on the surface of specimens.

After recording the morphologies of fatigue cracks, the eddy current was applied to the specimens with fatigue cracks under ambient conditions by a high frequency induction heating apparatus with a 60 KW rated power. The copper coils were connected with the high frequency induction heating apparatus and the specimens were fixed in the center of copper coils, as illustrated in Figure 2a. The copper coils were made from a copper tube 8 mm in diameter. The inner diameter of the copper coils was 45 mm, and the length of copper coils was 60 mm in order to cover the fatigue crack and cutting line area on the surface of the specimens. Different durations of the eddy current treatment were used in the two groups. Owing to the skin effect of eddy current, the eddy current density was the largest near the surface of the tubular specimen, so the outer surface temperature was the highest in the specimen, which was monitored by a FLIR infrared imager, as shown in Figure 2b. The maximum temperature of point O in the fatigue crack area (as shown in Figure 2b,c) was monitored to represent the temperature change of the fatigue crack. The temperature of points M, N, K, and L was measured at the same time; the points were symmetrically distributed on the two sides of point O along the circumferential direction as shown in Figure 2b,c. The four points nearly covered the neighboring area of the fatigue crack, so the average temperature of points M, N, K, and L was calculated to represent the temperature of the metal matrix around point O.

## 3. Results and Discussion

### 3.1. Axial Crack

**Figure 3:**
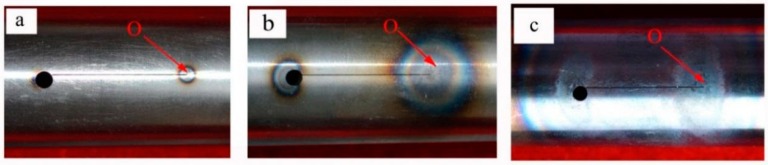
Photographs of specimens in group A: (**a**) A1; (**b**) A2; (**c**) A3.

**Figure 4:**
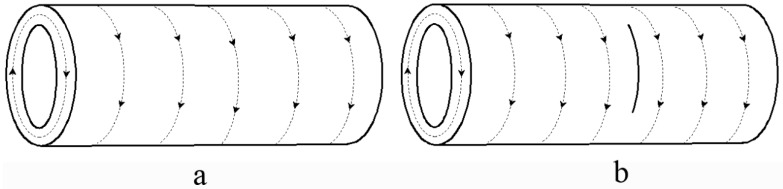
Schematics of eddy current within tubular specimens: (**a**) specimen without cracks; (**b**) specimen with axial cracks.

Table 1 presents the temperature in the vicinity of the fatigue cracks during the eddy current treatment. It can be seen that the maximum temperature at point O of tubular specimens in group A increased from 226 °C to 781 °C with the increase of eddy treatment duration from 1 s to 3 s, while the average temperature of point M, N, K, and L increased from 90 °C to 451 °C at the same time. During the eddy current treatment, the temperature at point O was nearly the maximum temperature in the monitored area, which could also be verified by the pictures in Figure 3. As shown in Figure 3, the surface oxidation area was expanded as a result of the increase of the maximum temperature of point O with the increase of the heating time. All the surface oxidation areas were centered around the end of the cutting lines where the fatigue cracks initiated, indicating that the maximum temperature on the surface of the specimen appeared in the fatigue crack area. The local oxidation area may be explained by the influence of eddy current induced by the copper coil during the eddy current treatment process. According to Faraday’s law of electromagnetic induction [19], the eddy current flows along a circular loop within the specimens under the ideal conditions, as illustrated in Figure 4a. When a crack appears along the axial direction, it disturbs the distribution of eddy current lines, as shown in Figure 4b. In this case, the eddy current lines detour around the crack tip and become denser on the crack tip, leading to the pronounced increase of the eddy current density there. The higher density of eddy currents produce more heat, resulting in much a higher temperature in the crack area.

**Figure 5:**
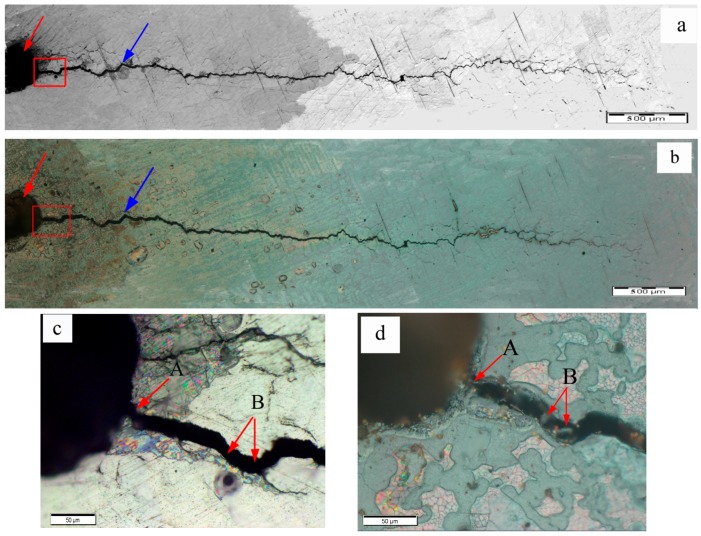
Optical microscope images for fatigue cracks of A1: (**a**) fatigue crack before the eddy current treatment; (**b**) fatigue crack after the eddy current treatment; (**c**) crack initiation zone of specimen A1 before the eddy current treatment; (**d**) crack initiation zone of specimen A1 after the eddy current treatment.

**Figure 6:**
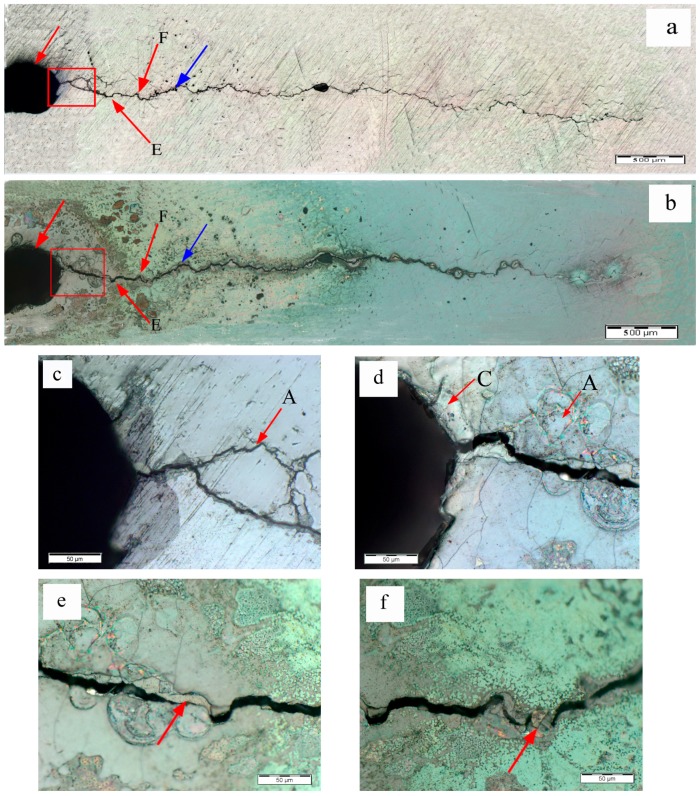
Optical microscope images for fatigue cracks of specimen A2: (**a**) fatigue crack before the eddy current treatment; (**b**) fatigue crack after the eddy current treatment; (**c**) crack initiation zone of specimen A2 before the eddy current treatment; (**d**) crack initiation zone of specimen A2 after the eddy current treatment; (**e**) marked area E in Figure 6b; (**f**) marked area F in Figure 6b.

**Figure 7:**
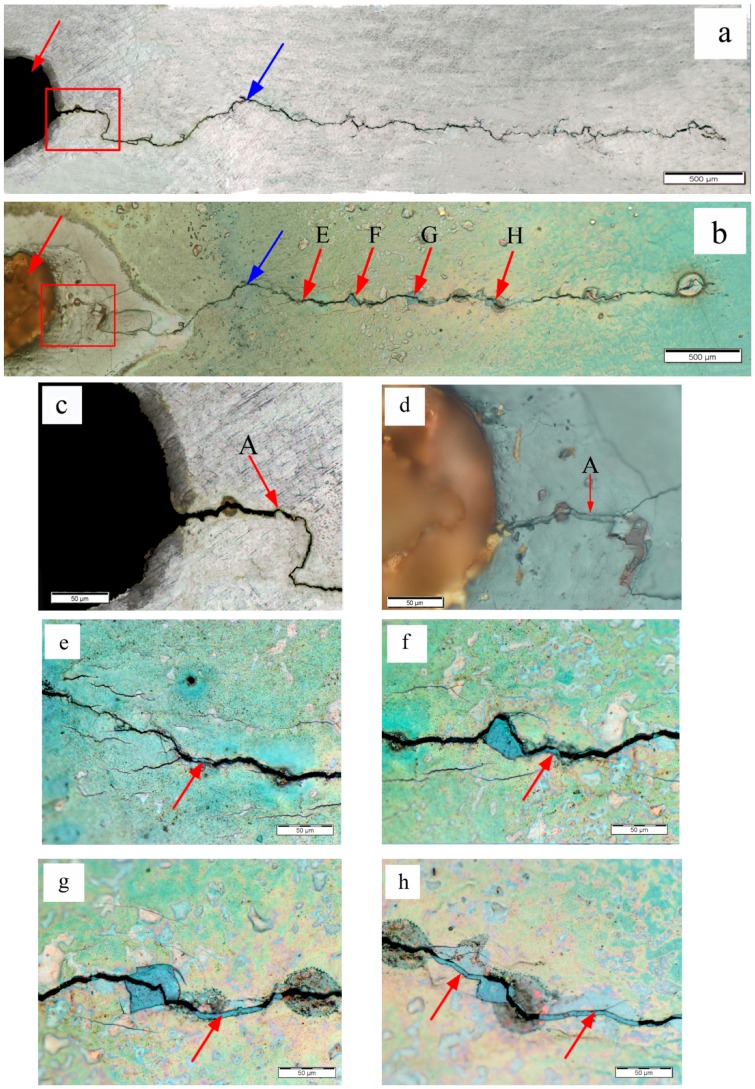
Optical microscope images for fatigue crack of specimen A3: (**a**) fatigue crack before the eddy current treatment; (**b**) fatigue crack after the eddy current treatment; (**c**) crack initiation zone of specimen A3 before the eddy current treatment; (**d**) crack initiation zone of specimen A3 after the eddy current treatment; (**e**) marked area E in Figure 7b; (**f**) marked area F in Figure 7b; (**g**) marked area G in Figure 7b; (**h**) marked area H in Figure 7b.

Further details were studied on the fatigue crack areas of the specimens. The optical micrographs of fatigue cracks of specimens A1, A2, and A3 before and after eddy current treatment are shown in Figure 5, Figure 6 and Figure 7, respectively. The left, wider crack is the cutting line (as the red arrow indicates) and the narrow crack (as the blue arrow indicates) is the fatigue crack connected with the cutting line. The surface oxidation area is also visible in Figure 5, Figure 6 and Figure 7. However, the lengths of the fatigue cracks remained almost the same after eddy current treatment when they were observed in the large scale, as shown in Figure 5a,b, Figure 6a,b and Figure 7a,b. The local areas of specimen A1 were further observed in small scale to confirm the difference between fatigue cracks before and after the eddy current treatment. The optical micrographs of the crack initiation zone of specimen A1 before and after the eddy current treatment are shown in Figure 5c,d respectively, which are magnified pictures of the areas in the red rectangles in Figure 5a,b. The crack initiation zone was the connecting zone of the cutting line and the fatigue crack. By comparing the Figure 5c,d, it could be seen that the crack initiation area was not filled, but that bridging appeared between two sides of the crack in the marked area A, which was very close to the position where the fatigue crack initiated. In addition, the marked area B near the marked area A also had a tendency to bridge, as shown in Figure 5d.

The fatigue cracks in specimens A2 and A3 exhibited a higher degree of healing than in specimen A1 as shown in Figure 6 and Figure 7. The local areas of specimen A2 were also observed in Figure 6. It is clear that the crack initiation zone was filled in marked area A shown in Figure 6d, which is the magnified picture of the area in the red rectangle in Figure 6b. Furthermore, the areas farther from the cutting line also exhibited the healing effect. The fatigue cracks in marked area E and F in Figure 6b were also partly filled, as illustrated in Figure 6e,f, respectively. Moreover, it was noted that the marked area C at the very beginning of the fatigue crack may have melted during the eddy current treatment, as depicted in Figure 6b,d, since the surface morphology in this area was quite different from the neighboring areas.

The specimen A3 exhibited the highest degree of healing, as shown in Figure 7. The Figure 7c,d are magnified pictures of the areas in the red rectangles in Figure 7a,b, respectively. It can be seen that the crack was completely filled and healed in the crack initiation zone after the eddy current treatment, and the filling level of specimen A3 in the crack initiation zone was much higher than that of specimen A2 and specimen A1. Furthermore, more filled areas were detected, as illustrated in Figure 7b. The fatigue cracks in marked areas E, F, G and H were also partly healed, as depicted in Figure 7e–h respectively. The marked area H was the farthest filled area from the cutting line in Figure 7b, and the distance between the marked area H and cutting line was much longer than the distance between the cutting line and marked area F in Figure 6b, which was the farthest filled area from the cutting line in Figure 6b. In addition, the local melting probably occurred in the crack initiation zone of specimen A3, which showed different color and morphology from the neighbor area after the eddy current treatment. Moreover, this local melting area was larger than the marked area C which may melt in Figure 6d.

**Figure 8:**
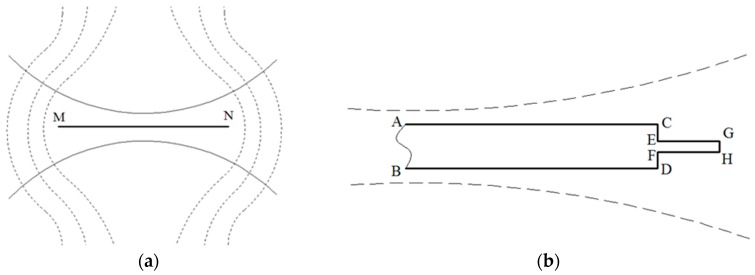
Schematics of eddy current around a crack: (**a**) whole crack; (**b**) the right end of whole crack.

The crack healing in the connecting zones of cutting lines and fatigue cracks in Figure 5, Figure 6 and Figure 7 was probably caused by air gap voltage breakdown. As mentioned above, the eddy current detoured around the crack tip because of the existence of fatigue cracks. Figure 8 shows the voltage distribution around the crack during the eddy current treatment. In Figure 8a, the line MN stands for the whole crack consisting of the cutting line and fatigue crack, and the dashed line and solid line stand for the eddy current line and the voltage equipotential line, respectively. The voltage equipotential lines were in arc shape, perpendicular to the eddy current lines according to the Faraday’s law of electromagnetic induction [18]. As a result, the neighboring voltage equipotential lines were very close at the center of the crack, causing higher electric field intensity in this area. In order to describe the eddy current distribution around the fatigue crack, the magnified schematic of the right end of the whole crack in Figure 8a is shown in Figure 8b. The profiles of the cutting line and fatigue crack are simplified for the convenience of analysis, wherein the wide rectangle ABCD stands for the right end of the cutting line, the narrow rectangle EFGH stands for fatigue crack, and the connecting zone of the cutting line and fatigue crack is close to EF. In addition, the dashed line stands for the voltage equipotential line. In fact, the length of fatigue crack was much shorter than the length of the cutting line, and the width of fatigue crack was also much smaller than that of the cutting line. According to the Paschen Law [19]:

(1) $$U=\frac{BPd}{\ln\left[\frac{APd}{\ln\left(1+\frac{1}{\gamma }\right)}\right]}$$

where *U* was the breakdown voltage, *P* was the gas pressure, *d* was the gap distance between the two sides of the crack, γ was the secondary electron emission coefficient at the cathode, A was the saturation ionization in the gas at a particular *E/p* (electric field/pressure), and *B* was related to the excitation and ionization energies. Since the parameters *A, B,* and γ are roughly constant over a restricted range of *E/P* for a given gas in a stable atmospheric pressure environment, the breakdown voltage *U* of the air gap was only related to the gap width *d*. The wider the gap *d* is, the higher breakdown voltage *U* is needed. Although the cutting line was in the middle of the copper coil along the axial direction and withstood the highest voltage, the width of the cutting line was almost ten times of the width of fatigue crack, which was too wide for the cutting line to be broken down. Hence, the breakdown of the air gap was more prone to take place in the fatigue crack area (EFGH) rather than the cutting line area (ABCD). Moreover, the breakdown appeared first in the connecting zone of the cutting line and fatigue crack (close to EF) where the fatigue crack initiated, since the zone was closer to the center of the copper coil and withstood the highest voltage compared to other fatigue crack zones. The breakdown effect triggered the rapid temperature rise in the connecting zone, leading to melting and filling of metal in this area. Longer eddy treatment duration may contribute to more heat accumulation, and thus more metal would melt and fill the crack, resulting in a higher healing level, which was verified through the comparison of specimen A1, A2, and A3. Therefore, the area which may melt in the crack initiation zone of specimen A3 became larger than the area in specimen A2, and more filled areas appeared in specimen A3.

**Figure 9:**
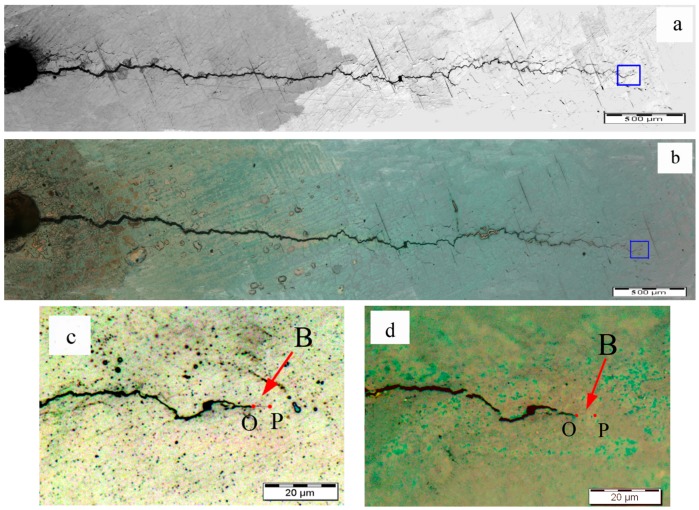
Optical microscope images for fatigue cracks of A1: (**a**) fatigue crack before the eddy current treatment; (**b**) fatigue crack after the eddy current treatment; (**c**) crack tip area of specimen A1 before the eddy current treatment; (**f**) crack tip area of specimen A1 after the eddy current treatment.

**Figure 10:**
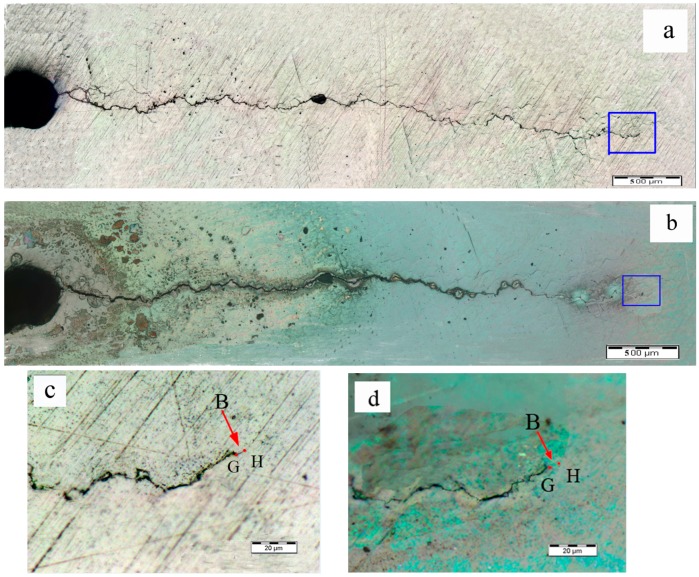
Optical microscope images for fatigue cracks of A2: (**a**) fatigue crack before the eddy current treatment; (**b**) fatigue crack after the eddy current treatment; (**c**) crack tip area of specimen A2 before the eddy current treatment; (**d**) crack tip area of specimen A2 after the eddy current treatment.

**Figure 11:**
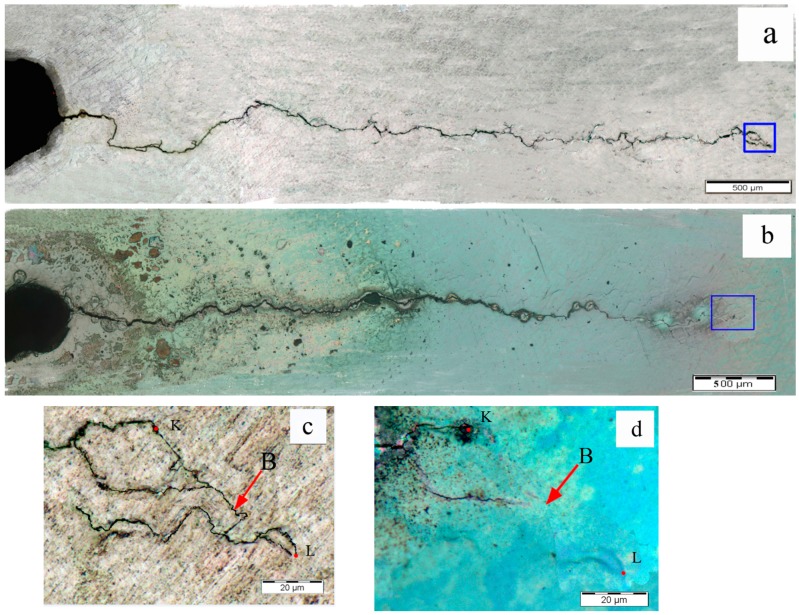
Optical microscope images in local area of specimen A3: (**a**) fatigue crack before the eddy current treatment; (**b**) fatigue crack after the eddy current treatment; (**c**) crack tip area of specimen A3 before the eddy current treatment; (**d**) crack tip area of specimen A3 after the eddy current treatment.

The connecting zone of the cutting line and fatigue crack was not the only area healed. The fatigue crack tip area was also healed after the eddy current treatment, as can be seen in Figure 9, Figure 10 and Figure 11. The Figure 9c,d are the magnified pictures of fatigue crack tip areas in the blue rectangles in Figure 9a,b. When observed in large scale in Figure 9a,b, there was no obvious difference in the crack tip before and after the eddy current treatment. However, the marked area B in Figure 9a zoomed in Figure 9c presented the microcrack OP existing in this area before the eddy current treatment, while it disappeared after the eddy current treatment, as shown in Figure 9d, indicating that the crack tip area was healed after the eddy current treatment. The similar situation also occurred in the crack tip of specimen A2 and specimen A3. The Figure 10c,d are the magnified pictures of fatigue crack tip areas in the blue rectangles in Figure 10a,b, and the Figure 11c,d are the magnified pictures of fatigue crack tip areas in the blue rectangles in Figure 11a,b. Through comparing the crack tips before and after eddy current treatment (see the marked areas B in Figure 10c,d and Figure 11c,d), the microcrack GH in the fatigue crack tip of specimen A2 and the micro-crack KL in the fatigue crack tip of specimen A3 were healed after eddy current treatment. Because the profiles of the crack tips were quite different in different specimens, it was hard to quantitatively compare the healing lengths of crack tips. In order to simplify the calculation of crack length, the straight-line lengths of OP, GH, and KL were used to represent the healing lengths. The specimen A3 exhibited the longest length of crack healing (65.6 μm) in the crack tip area, while the specimen A2 showed only a slightly longer length of crack healing (7.5 μm) in the crack tip area than the specimen A1 (7.1 μm), which indicated that the healing degree increased non-linearly with the increase of time duration in the present study.

The mechanism for the crack healing in the crack tip caused by the eddy current treatment was not very clear until now; the possible reasons are compressive stress and local melting due to the detouring of the eddy current. When the eddy current flowed around the crack tip, it gathered in the crack tip area, leading to much higher current density in the crack tips than in other areas. The higher current density produced more heat and resulted in a significant rise in temperature, which causes metal melting in local areas and contributes to crack healing. In addition, the detouring of the eddy current around the crack tip made the temperature increase more quickly than in other areas, so a nonsynchronous change of temperature rise and thermal expansion would be generated there [18]. Since the thermal expansion rate of metal material is proportional to their temperature, the thermal expansion in the vicinity of the crack tip would be much higher than the thermal expansion in areas far from the crack tip, thus the thermal expansion of the crack tip would be suppressed by the surrounding area. Therefore, there was thermal stress around the crack tip due to the compressive force exerted by the surrounding area. The maximum theoretical thermal stress due to a nonsynchronous change of temperature rise and thermal expansion was given by Hooke’s law [20]:

(2) $$\sigma_{max}=E\varepsilon$$

where *E* is the Young’s modulus and ε is the strain of material in the crack tip area. Here the strain ε of material in the crack tip area was ascribed to the thermal expansion of material, so the strain ε could be given by following equation:

(3) $$\varepsilon =\frac{\Delta L}{L}=\alpha \Delta T$$

where α is the thermal expansion coefficient of the specimen and Δ*T* is the temperature difference between the crack tip area and neighboring area of the fatigue crack. For the approximate analysis, the maximum temperature of the crack tip was denoted by the maximum temperature of point O in Figure 2c, while the average temperature of points M, N, K, and L was used to represent the temperature in the neighboring area of the fatigue crack. Therefore, the maximum theoretical thermal stress could be expressed as follows [7]:

(4) $$\sigma_{max}=E\alpha \Delta T$$

According to the experiment result, the temperature difference Δ*T* between the crack tip and neighboring area of the fatigue crack was about 136 °C in specimen A1. Referring to the thermal expansion coefficients of 1045 steel in Table 2, it gave the maximum theoretical thermal compressive stress σ_max_ ≈ 351 MPa, wherein the Young’s modulus *E* of 1045 steel was 210 GPa. For the specimens A2 and A3, we had Δ*T* ≈ 224 °C and 331 °C, respectively, and then the maximum theoretical thermal compressive stress σ_max_ ≈ 644 MPa and 861 MPa, respectively. Obviously, the thermal compressive stress around the crack tip increased gradually with the increased duration. Under the action of high compressive stress, the two sides of the crack would be pushed toward each other, and would close when their distance was small enough and the higher temperature existed around the crack, indicating that the crack tip had a high probability of being closed during the eddy current treatment. However, the crack healing in the crack tip area may not result only from the temperature change, it may also be related to the profile of the crack tip and the temperature of the metal matrix, etc., since different healing levels were achieved in specimens A1, A2 and A3. For example, the long treatment time would increase the temperature of the specimen matrix; but some studies found that high temperatures probably caused shrinkage and the smoothing of crack edges when the specimens were subject to hot isostatic pressing treatment, and thus led to the healing failure of the cracks [21].

**Figure 12:**
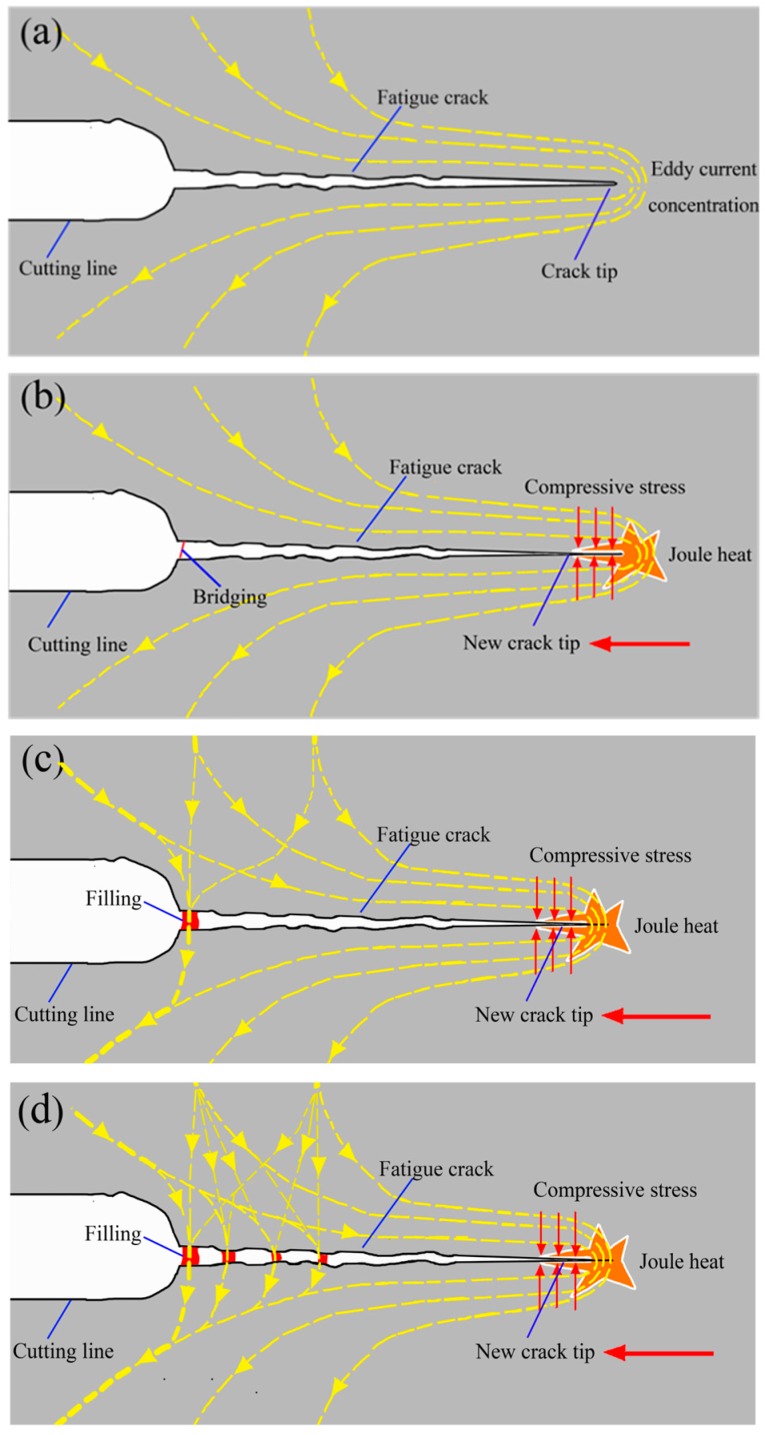
Schematics of the fatigue crack healing process. (**a**) the detour of eddy current; (**b**) the appearance of the compressive stress and the bridging; (**c**) the appearance of breakdown and crack tip healing; (**d**) continuous crack healing.

Based on the above analysis, the process of fatigue crack healing by eddy current treatment is summarized in Figure 12. The Figure 12a shows the beginning stage of eddy current treatment in which the eddy current (the dashed line) flowed around the fatigue crack and took a detour from the crack tip. As a result of joule heat generated around the crack tip, the local concentration of eddy current around the fatigue crack tip caused the closure or healing of the crack tip and subsequent movement of the new crack tip (as the red arrow indicates) toward the left side under compressive stress, as shown in Figure 12b. On the other hand, the voltage breakdown contributed to the bridging in the connecting zone of the cutting line and the fatigue crack in Figure 12b. As the treatment duration increased, the area where the bridging took place was filled due to the diffusion of metal material, as depicted in Figure 12c. After that, part of the eddy current that was detouring around the crack tip before would flow through the filled area, which decreased the current density detouring around the crack tip. Hence, the compressive stress around the new crack tip may be reduced because of the heat reduction in this area caused by the detour of the eddy current, which decreased the possibility of the new crack healing. However, the eddy current flowing through the filling area created a large amount of heat in the filled area, and the accumulation of heat resulted in the filling of more areas near the previously filled area. Therefore, some narrow areas were filled successively, as illustrated in Figure 12d.

### 3.2. Radial Crack

**Figure 13:**
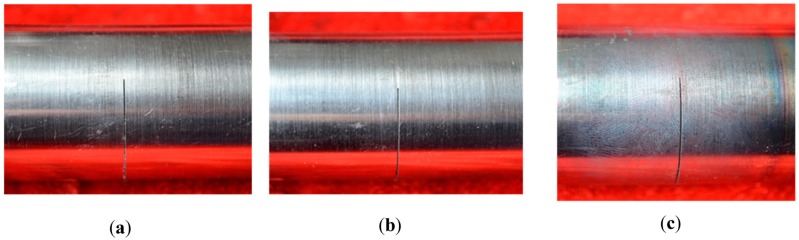
Photographs of specimens in group B after the eddy treatment: (**a**) B1; (**b**) B2; (**c**) B3.

**Figure 14:**
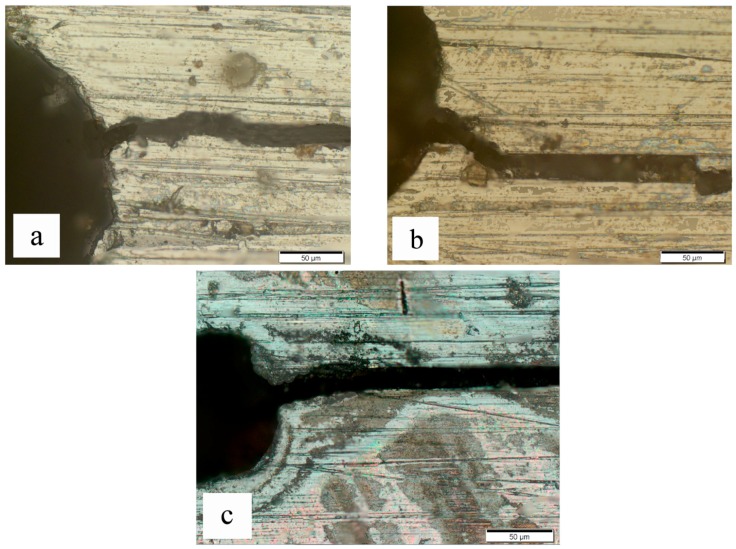
Optical micrographs of specimens B1, B2, and B3 after the eddy treatment: (**a**) B1; (**b**) B2; (**c**) B3.

The situation was totally different when the specimens in group B were subjected to the eddy current treatment. As can be seen from Table 1, the maximum temperature of point O of the specimens in group B increased from 155 °C to 496 °C with the increase of time duration from 1 s to 3 s, and the temperatures of point M, N, K, and L were always quite close to the temperature of point O. Although the treatment times for the two groups were the same, the maximum temperatures of the specimens in group B were much lower than that in group A, so no oxidation appeared on the surface of specimen B1 and B2, and slight oxidation uniformly appeared on the surface of specimen B3, as shown in Figure 13. Moreover, the crack area did not exhibit a higher oxidation degree than the neighboring areas. It indicates that the temperature was distributed almost evenly on the surface of tubular specimens during the eddy current treatment. In addition, the connecting zones of cutting lines and fatigue cracks were observed in Figure 14. It is clear that there was no healing in this area even when the maximum temperature reached 496 °C in specimen B3, which was even higher than the average temperature of specimens A1 and A2. Therefore, it could be speculated that the temperature of the metal matrix is not the decisive factor for crack healing of tubular specimens.

**Figure 15:**
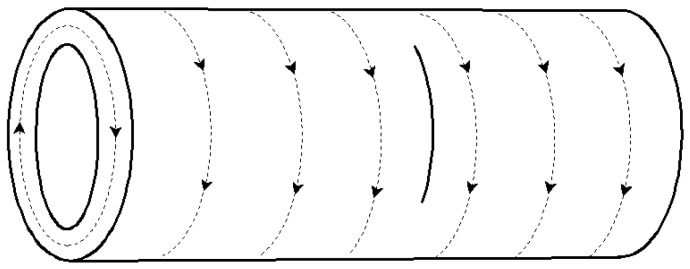
Schematic of the eddy current within specimens in Group B.

This phenomenon was also related to the eddy current distribution within the specimens in group B, as illustrated in Figure 15. When a crack along the radial direction appeared, it was not able to disturb the distribution of the eddy current lines along the hoop direction. The distribution of the eddy current lines was uniform and stable, and the joule heat induced by eddy current was almost uniformly distributed. Therefore, the eddy current did not detour around the fatigue crack, and the eddy current lines did not gather around the crack tip. In this case, no obvious heat concentration appeared on both sides of the fatigue crack, thus no crack healing occurred.

It is well known that the penetration depth of electrical current within conductors is influenced by the skin effect. For example, the skin depth in 1045 steel increases from 0.36 mm to 1.59 mm with the increase of magnetic field intensity from 10 A/mm to 280 A/mm at a frequency of 3000 Hz [23]. Therefore, the eddy current treatment may be more suitable for crack healing of thin-walled tube specimens. However, adjusting the frequency and density of eddy currents may increase the healing depth, since they could increase the skin depth and enlarge the eddy current density, respectively, in the deeper area. The increase of the healing depth should improve the fatigue crack growth resistance; this will be investigated in our future work.

## 4. Conclusions

The tubular specimens of 1045 steel with fatigue cracks along the axial direction and radial direction were treated by eddy current, and the influence of eddy current treatment duration on the healing of the fatigue cracks was evaluated in this study. The main conclusions can be summarized as follows:

1) The fatigue cracks along the axial direction were healed under eddy current treatment while the fatigue cracks along the radial direction were not healed. The crack tip area of the fatigue crack along the axial direction was healed, and the bridging appeared in the connecting zone of the cutting line and the fatigue crack simultaneously, followed by the filling in of the bridging areas. Consequently, more filled areas successively appeared near the previously filled areas.

2) The possible reason for the healing in the connecting zones of the cutting lines and the fatigue crack of the specimens with axial fatigue cracks was the voltage breakdown occurring in this area. Moreover, since the heat accumulation appeared in the connecting zones of the fatigue cracks and the cutting lines, some areas close to the cutting line were also filled, and the areas closer to the cutting line were more likely to be filled.

3) The healing areas appeared in the crack tips of the specimens with axial fatigue cracks, which resulted from the thermal compressive stress induced by the temperature difference between the crack tip area and its neighboring area due to the detouring of the eddy current in the crack tip.

4) The voltage breakdown in narrow gaps and the detouring of newly formed crack tips contributed to successive healing of the fatigue crack, which was enhanced with the increase of the treatment duration in the present study. 

5) When the specimens with fatigue cracks along the radial direction were subjected to the eddy current treatment, the eddy current within the specimens was evenly distributed and no detouring of the eddy current occurred around the fatigue cracks, therefore, the voltage breakdown and the local heating effects did not happen, and the fatigue cracks did not heal.

## Figures and Tables

**Table 1:** Treatment durations and surface temperatures of specimens.

Group	Specimen	Duration/Second	*T*_max_ of O/°C	*T*_max_ of M/°C	*T*_max_ of N/°C	*T*_max_ of K/°C	*T*_max_ of L/°C	*T*_average_ of M, N, K, L/°C
Group A	A1	1	226	103	112	75	68	90
	A2	2	376	205	187	110	105	152
	A3	3	781	552	572	356	324	451
Group B	B1	1	155	154	160	151	162	157
	B2	2	194	193	182	200	189	191
	B3	3	496	498	486	502	483	492

**Table 2:** Thermal expansion coefficients α of 1045 steel [22].

*T*/°C	20–200	20–300	20–400	20–500	20–600	20–700	20–800
α/(10^−6^mm/°C)	12.32	13.09	13.71	14.18	14.67	15.08	12.50
